# Osteogenesis-Related Behavior of MC3T3-E1 Cells on Substrates with Tunable Stiffness

**DOI:** 10.1155/2018/4025083

**Published:** 2018-11-01

**Authors:** Yingying Zhang, Yanghui Xing, Jian Li, Zhiqiang Zhang, Huiqin Luan, Zhaowei Chu, He Gong, Yubo Fan

**Affiliations:** ^1^Beijing Key Laboratory of Rehabilitation Technical Aids for Old-Age Disability and Key Laboratory of Intelligent Control and Rehabilitation Technology of the Ministry of Civil Affairs, National Research Center for Rehabilitation Technical Aids, Beijing 100176, China; ^2^Key Laboratory for Biomechanics and Mechanobiology of Ministry of Education, School of Biological Science and Medical Engineering, Beihang University, Beijing 100191, China

## Abstract

Osteogenic differentiation of cells has considerable clinical significance in bone defect treatment, and cell behavior is linked to extracellular matrix stiffness. This study aimed to determine how matrix stiffness affects cell morphology and subsequently regulates the osteogenic phenotype of osteogenesis precursor cells. Four PDMS substrates were prepared with stiffness corresponding to the elastic modulus ranging from 0.6 MPa to 2.7 MPa by altering the Sylgard 527 and Sylgard 184 concentrations. MC3T3-E1 cells were cultured on the matrices. Cell morphology, vinculin expression, and key osteogenic markers, Col I, OCN, OPN, and calcium nodule, were examined. The activity and expression level of Yes-associated protein (YAP) were evaluated. Results showed that cell spreading exhibited no correlation with the stiffness of matrix designed in this paper, but substratum stiffness did modulate MC3T3-E1 osteogenic differentiation. Col I, OPN, and OCN proteins were significantly increased in cells cultured on soft matrices compared with stiff matrices. Additionally, cells cultured on the 1:3 ratio matrices had more nodules than those on other matrices. Accordingly, cells on substrates with low stiffness showed enhanced expression of the osteogenic markers. Meanwhile, YAP expression was downregulated on soft substrates although the subcellular location was not affected. Our results provide evidence that matrix stiffness (elastic modulus ranging from 0.6 MPa to 2.7 MPa) affects the osteogenic differentiation of MC3T3-E1, but it is not that “the stiffer, the better” as showed in some of the previous studies. The optimal substrate stiffness may exist to promote osteoblast differentiation. Cell differentiation triggered by the changes in substrate stiffness may be independent of the YAP signal. This study has important implications for biomaterial design and stem cell-based tissue engineering.

## 1. Introduction

Medical implants are widely used in clinical treatment. The surface properties of these implants, such as roughness, topography, energy, and chemistry, vary substantially. All of these properties affect bone-to-implant contact [1, 2], and several studies have shown that their effects are partly caused by the regulation of the cell osteoblastic differentiation during bone healing [3]. Cells are sensitive to material properties of substrate, such as stiffness, surface roughness, and energy. Considerable amount of evidence suggests that substrate properties play a role in inducing stem cell differentiation into osteoblasts [4, 5], but whether osteogenic differentiation is mediated by specific stiffness is unclear.

Researchers have developed many materials systems to probe the interactions between mechanical stiffness and cell behaviors. In previous cases, cells were cultured on gels with elastic moduli in the range of ~0.1 kPa to ~100 kPa [6–9]. For instance, on the stiffer matrices (25-40 kPa) that mimic the cross-linked collagen of osteoids [10], MSCs remarkably upregulate the osteogenesis marker compared with cells on softer matrices (0.1-17 kPa) [6]. Engler et al. [6] found that the elastic modulus of the bone collagenous osteoid precursors is ~100 kPa. Adipose stem cells cultured on PDMS-based matrices with stiffness ranging from 1.4 kPa to 134 kPa show effective osteogenic differentiation induction on the stiffest matrix because matrix with the stiffness of 134 kPa matches that of cancellous bone [11]. It was also demonstrated that cells are sensitive to substrate elastic modulus ranging from 100 kPa to 1 MPa [12]. However, the stiffness of human tissues and organs vary widely, and most orthopedic polymer implants possess moduli in the megapascal to gigapascal range [13]. Numerous studies were performed on polymer and metal substrates with lower or higher moduli range than that of native bone, where such biomaterials generally are placed. Additionally, stiffness variations exist in bone because the component of bone and degree of mineralization are unstable. The relationship between substrates with a tunable modulus and osteoblast response needs further study.

Substrates stiffness regulates cell differentiation primarily via focal adhesion (FA). FAs vary with substrate stiffness [6]. As a key transmitter, FA allows extracellular biophysical cues transforming into intracellular signals, which can influence cytoskeletal structure and mediate cell biology [11, 14]. Vinculin is the key FA protein, and enhanced vinculin level upregulates cellular functions such as proliferation, spreading, and differentiation [15, 16]. Yes-associated protein (YAP) is a sensor of mechanical cues instructed by the cellular microenvironment [17]. YAP is also one of the nuclear relays of mechanical signals exerted by ECM properties and cell shape [18]. Cytoskeleton plays an important role in mechanical stimuli transduction to the Hippo pathway [18, 19]. YAP is required in mediating the cellular responses to matrix stiffness because YAP depletion inhibits osteogenic differentiation [18]. However, intracellular signals generated following stiffness stimulation are complicated, and the pathway is not well understood.

In the present study, a series of polymer substrates with varying stiffness was fabricated by blending two commercially available PDMS types, that is, Sylgard 527 and Sylgard 184 [12]. This method is ideal in altering the elastic modulus without changing the surface roughness and energy. The stiffness range we considered (0.6-2.7 MPa) was much larger than those reported. Cell culture experiments were conducted to identify how MC3T3-E1 cells respond to a range of substrate stiffness by assessing morphology, vinculin expression, production of certain osteogenic markers, and YAP activity/protein level. This study is expected to provide important insights into the design of scaffold for bone tissue engineering.

## 2. Materials and Methods

### 2.1. Preparation of Substrates with Tunable Stiffness

Tunable substrates were fabricated based on a previous established protocol [12]. Briefly, Sylgard 184 and Sylgard 527 (Dow Corning, USA) were blended by mass ratios. The different mass ratios of the Sylgard 184 to Sylgard 527 are 1:0, 5:1, 1:1, and 1:3 in the order of decreasing stiffness. Each blend was mixed by first preparing pure Sylgard 527 and 184 as follows. Sylgard 184 was mixed following the manufacturer's instructions in a 10:1 base to curing agent ratio and then defoamed. Sylgard 527 was prepared by mixing equal weights of part A and part B in the same mixing and defoaming cycle. Once mixed, the prepolymer was either poured into 12-well cell culture plates to create ~3 mm thick films for cell culture or poured into 100 mm dish to create ~6 mm thick films for mechanical testing. All substrates were cured for 4 h at 65°C and then stored at room temperature. Prior to cell seeding, substrates were sterilized by immersion in ethanol overnight and then exposed to ultraviolet light for 70 min. Subsequently, the samples were incubated in 20 *μ*g/mL fibronectin (FN; BD Bioscience, USA) solution overnight at 4°C. The substrates were rinsed three times with phosphate-buffered saline (PBS) and ready for seeding MC3T3-E1 cells. The elastic modulus for each substrate was measured with a biomechanical testing machine (TA Electro Force 3200, USA) under contact load at a strain rate of 0.01 mm/s. Six samples from each preparation were analyzed.

### 2.2. Cell Culture

The preosteoblasts, MC3T3-E1, were prepared for all subsequent experiments. Cells were cultured in *α*-minimal essential medium (*α*-MEM, Hyclone, USA) supplemented with 10% FBS (Hyclone, USA) and 1% penicillin-streptomycin (Life Technologies, USA). Cells were plated on the FN-coated substrates at a density of 1×10^4^ cells/cm^2^ and cultured in the growth medium supplemented with osteogenic factors (10 nM *β*-glycerol phosphate, 50 *μ*g/mL ascorbic acid; Sigma, St. Louis, USA). The differentiation-inducing medium was changed every other day.

### 2.3. Immunostaining

After the cells were cultured on each substrate for 24 h, they were washed with prewarmed PBS three times (10 min per wash), then fixed in 4% paraformaldehyde for 15 min, washed three times with PBS, and permeabilized with 0.2% Triton X-100 for 10 min at room temperature. Then, the cells were blocked with 1% BSA in PBS (Sigma, St. Louis, USA) for 30 min and incubated with phalloidin (Molecular Probes) solution and monoclonal anti-vinculin antibody (Sigma, St. Louis, USA) as primary antibodies for 60 min at 37°C. After incubation, the samples were washed three times and followed by 60 min incubation with FITC-conjugated rabbit anti-mouse antibody (Zhongshan, Beijing, China). Nuclei were stained with Hoechst 33258 (Sigma, St. Louis, USA). Finally, the substrates with cells were examined by confocal microscopy (Leica Microsystems GmbH, Germany).

### 2.4. Protein Isolation and Western Blot Analysis

The cells on each substrate were lysed in buffer supplemented with the protease inhibitor. Proteins were quantified, and then equal amounts of protein lysates (~20 *μ*g) were separated by electrophoresis on 10% polyacrylamide gels and transferred onto a nitrocellulose filter membrane. Membranes were blocked, followed by overnight incubation at 4°C with antibodies for Col I (Bioss, Beijing, China), osteocalcin (OCN, Bioss, Beijing, China), osteopontin (OPN, Boster, Wuhan, China), YAP (ABclonal, MA, USA), and ACTB (Cell Signaling, USA). After being washed for three times, the primary antibody binding was detected using secondary antibodies for 60 min at room temperature on the next day. Immunoreactive bands were visualized by enhanced ECL (Pierce, Rockford, USA) and Tanon Image Analysis System (Tanon, Beijing, China). Densitometry analysis of the bands was performed by Image J software (NIH).

### 2.5. Alizarin Red Staining for Calcium and Quantitative Analysis

To detect the mineralized matrix, the cells cultured on each substrate for 4 weeks were fixed with 70% ethanol for 10 min and stained with an appropriate amount of 0.1% (w/v) alizarin red (pH 4.2) solution for 30 min at room temperature. After washing, photographs were taken. Then, cetylpyridinium chloride (10%, pH 7.4) solution was added to the dry samples, followed by the destaining procedure for 10 min. Samples were finally transferred to a 96-well plate, and the absorbance at 562 nm was measured with a microplate reader (SpectraMax i3, Molecular Devices, USA).

### 2.6. Gene Expression Analysis

Total RNA was extracted by using TRIzol reagent (Invitrogen, CA, USA), quantified by NanoDrop 2000 (Thermo Fisher Scientific, Wilmington, DE, USA). The first-strand cDNA was synthesized in a reaction volume of 20 *μ*L containing 1 *μ*g total RNA using a PrimeScript RT reagent Kit (TaKaRa, Japan). The following primers (synthesized by Invitrogen) were used in Q-PCR reactions: GAPDH: 5′- ACCTGCCAAGTATGATGAC-3′ and 5′- CTGTTGCTGTAGCCGTAT-3′; RAP2, 5′- GGTTGCTTAGTCGCTTAGT-3′ and 5′- CATCTGATTGGCTGAGGATA-3′; Q-PCRs were then carried out in a reaction volume of 20 *μ*L on an iQ5 Cycler realtime PCR system (Bio-Rad, Hercules, CA, USA) to determine the relative expression of RAP2. The PCR reaction mixture consisted of 1 *μ*L cDNA, 0.2 *μ*M each primer, and 10 *μ*L SYBR Green PCR Master Mix (TaKaRa, Japan). The thermal profile consisted of an initial 10 min at 95°C, followed by 40 cycles of 95°C for 15 s and 60°C for 1 min. The gene expression results were normalized to that of an internal control (GAPDH), and the comparative Ct method was used to calculate the relative mRNA levels. Primers were synthesized by Invitrogen.

### 2.7. Statistical Analysis

All tests were reported as mean ± SEM. Statistical analysis was performed using GraphPad Prism. Two groups were compared using unpaired Student's* t*-test.* p* < 0.05 was considered statistically significant.

## 3. Results

### 3.1. Mechanical Properties of the Substrates

**Figure 1:**
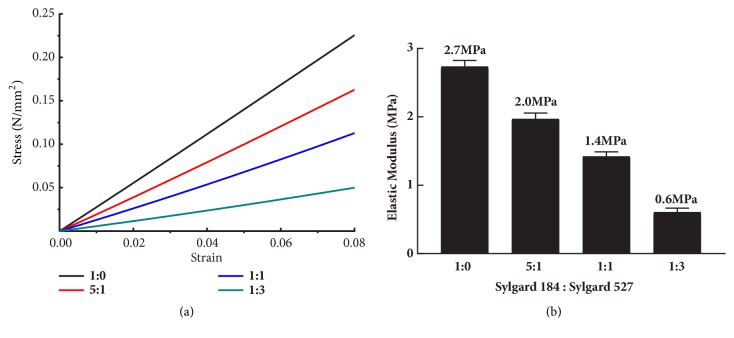
Substrates with varying stiffness were fabricated by tuning the ratios of Sylgard 184 mixed with Sylgard 527 (from 1:0, 5:1, and 1:1 to 1:3). (a) Representative stress–strain curves for four different PDMS formulations measured by biomechanical testing. (b) Elastic modulus of the four different substrates obtained from the load–distance curve.

Blending Sylgard 527 and Sylgard 184 by different mass ratios can tune the mechanical properties of substrates [12]. Hence, in this study, four substrates with different elastic moduli were prepared. Representative stress–strain curves in biomechanical test demonstrated that the four substrates possessed different elastic moduli (Figure 1(a)). The curves were linear under the detected strain range. The average value of elastic modulus was calculated by the slope of these curves. Increasing the ratio of Sylgard 527 relative to Sylgard 184 decreased the elastic modulus from ~2.7 MPa to ~0.6 MPa (Figure 1(b)). We will subsequently refer to the substrates with different elastic moduli by the mass ratio to simplify our terminology, specifically Sylgard 184:527 = 1:0, 5:1, 1:1, 1:3.

### 3.2. Cell Morphology on Substrates with Different Stiffness

**Figure 2:**
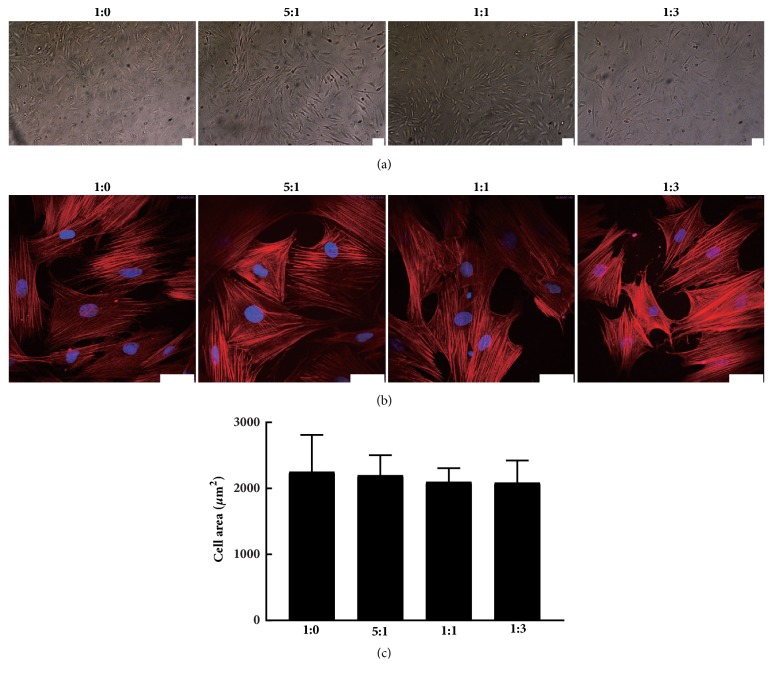
Morphological appearance of MC3T3-E1 plated on substrates with different stiffness. (a) Phase contrast images of cells cultured on the prepared substrates at 24 h after seeding (scale bar: 100 *μ*m). (b) Confocal immunofluorescent images of actin filaments (phalloidin) and nucleus (hoechst) in cells on different substrates (scale bar: 50 *μ*m). (c) MC3T3-E1 spread area on substrates.

After 24 h of culture, MC3T3-E1 cells adhering to different substrates were observed. Initial observation by inverted microscope confirmed that cells tightly adhered to all substrates and grew well (Figures 2(a) and 2(b)). However, no distinct difference was observed on cell shape or growth in response to the different substrates (Figure 2(b)). Phalloidin staining of F-actin also showed that all cells reached their maximum spreading areas (Figure 2(b)). Nevertheless, no correlation was observed between cell spreading area and substrate stiffness (Figure 2(c)). Hence, we did not observe morphological changes in MC3T3-E1 on all tested surfaces.

### 3.3. Cell FAs Regulated by Substrates Stiffness

**Figure 3:**
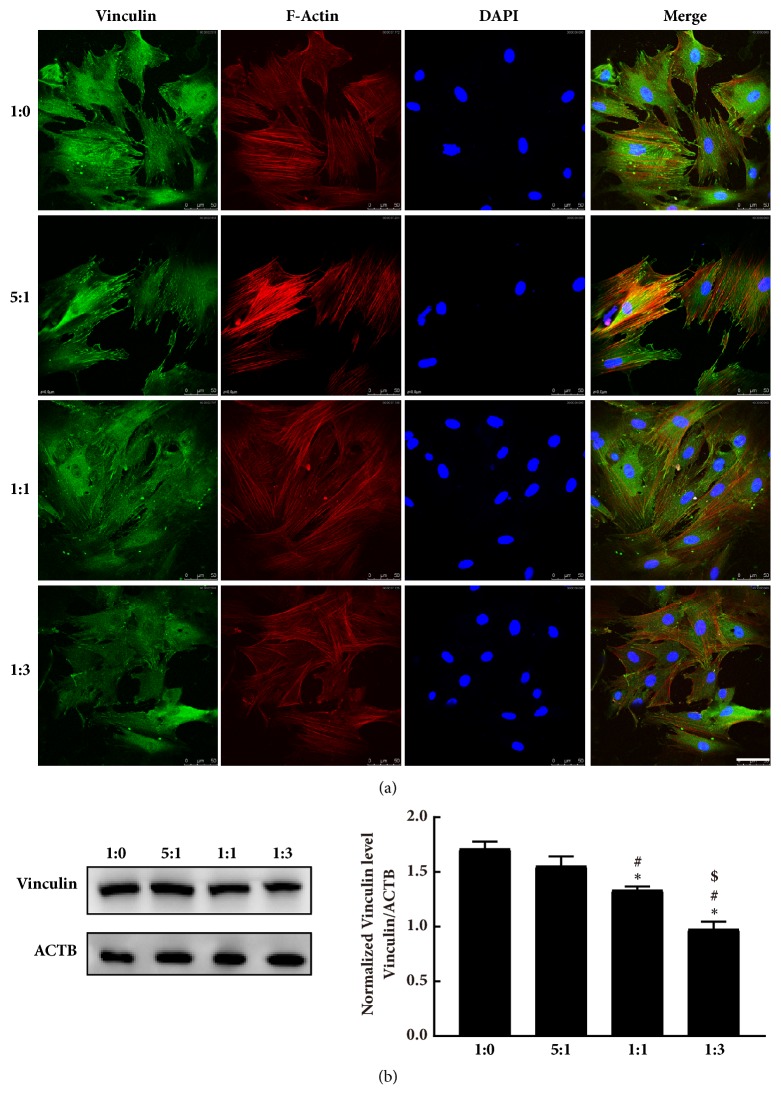
Focal adhesion was regulated by substrate stiffness. (a) Immunofluorescent images of vinculin in MC3T3-E1 plated on different substrates (ranging from 1:0 to 1:3, elastic modulus ranging from 2.7 MPa to 0.6 MPa) (scale bar = 50 *μ*m). (b) The protein expression level of vinculin detected by western blotting and quantitative analysis. Vinculin results were normalized to ACTB. *∗p* < 0.05 vs. 1:0,* #p* < 0.05 vs. 5:1, $*p*< 0.05 vs. 1:1.

FA is a transmitter that may influence cytoskeletal structure and mediate cell biology [14]. The key FA protein, that is, vinculin, was also observed by immunofluorescence in the present study. The images of F-actin distribution showed that actin polymerization and FA were stiffness-dependent (Figure 3(a)). On rigid substrates (ratio of 1:0), FA showed strong rod-like structure and was more along the cell margins. As matrix stiffness decreased (ratio ranging from 1:0 to 1:3), vinculin showed short and dense distribution. Similar to these observations, western blotting results also showed that vinculin expression decreased clearly as the substrates became softer (Figure 3(b)).

### 3.4. Osteogenic Differentiation of MC3T3-E1 Was Increased as the Substrates Became Softer

**Figure 4:**
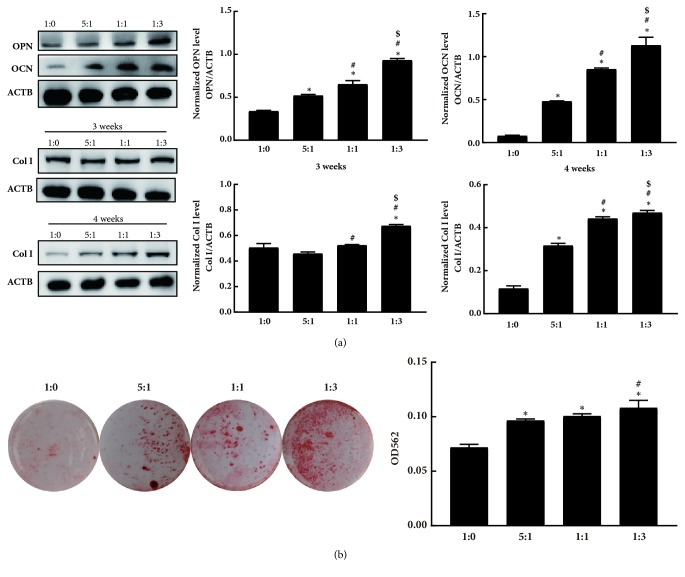
Osteogenesis capacity of MC3T3-E1 was altered by substrate stiffness. (a) The expression of osteoblast markers OPN, OCN, and Col I measured by western blot analysis after 3 and 4 weeks of culture on different substrates. (b) Alizarin red staining of mineral nodule formation on the substrates of cells cultured for 4 weeks and quantitative analysis of dissolved color by reading the absorbance at 562 nm. *∗p* < 0.05 vs. 1:0,* #p* < 0.05 vs. 5:1, $*p*< 0.05 vs. 1:1.

To explore the progress of osteogenic differentiation on various surfaces, we evaluated the OPN, OCN, and Col I protein levels by western blot analysis, which are all well-known osteogenic differentiation markers. Consistently, OPN and OCN proteins were significantly increased in MC3T3-E1 cultured on soft matrices compared with stiff matrices (Figure 4(a)). Col I expression also increased on 1:3 ratio matrix relative to the 1:0 matrix, although the level decreased after 4 weeks for all surfaces, as indicated in Figure 4(a). Mineralization of MC3T3-E1 on substrates was also confirmed by alizarin red staining (Figure 4(b)). After incubating cells on the surfaces for 4 weeks, cells cultured on the 1:3 ratio matrices had more nodules than those on other matrices. Then, the stained samples underwent destaining procedure. The results also showed that softer substrates presented higher mineralization reaction of MC3T3-E1 than their stiffer counterparts (Figure 4(b)).

These results suggested that the osteogenic differentiation of MC3T3-E1 cells was favored for soft matrix in substrates with elastic modulus ranging from 0.6 MPa to 2.7 MPa.

### 3.5. YAP Expression Was Decreased as the Substrates Became Softer

**Figure 5:**
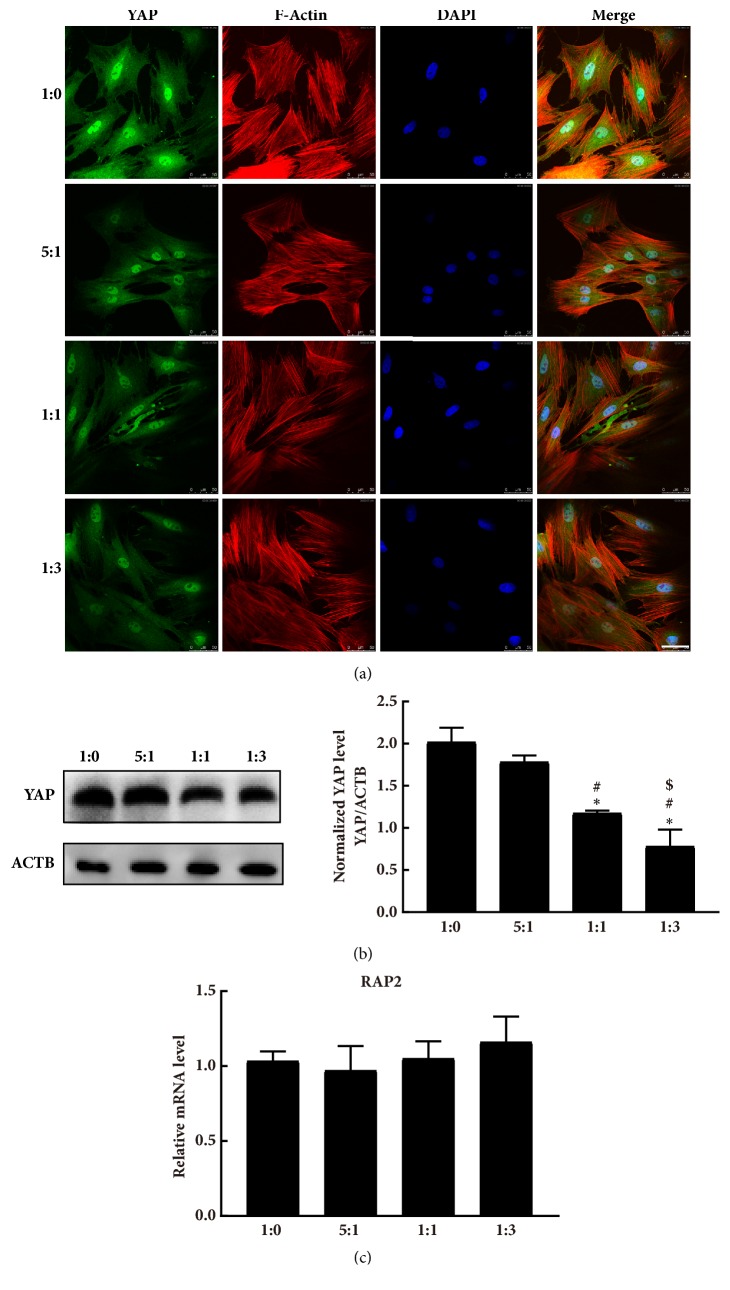
MC3T3-E1 cell response to substrates with tunable stiffness observed in YAP protein level. (a) Immunofluorescent images of YAP in MC3T3-E1 plated on different substrates (ranging from 1:0 to 1:3, elastic modulus ranging from 2.7 MPa to 0.6 MPa) (scale bar = 50 *μ*m). (b) YAP protein expression detected by western blotting and quantitative analysis by densitometry. YAP results were normalized to ACTB. *∗p* < 0.05 vs. 1:0,* #p* < 0.05 vs. 5:1, $*p*< 0.05 vs. 1:1. (c) Quantitative RT-PCR analysis of RAP2 in MC3T3-E1 cultured on different substrates (n=3). All results were normalized against GAPDH, and the comparative threshold cycle method (^ΔΔ^Ct) was used to determine the relative mRNA levels. Data are shown as fold change in relation to the gene expression on 1:0 substrate.

We monitored YAP activity and protein expression level in MC3T3-E1 cells grown on our fibronectin-coated PDMS with varying stiffness. The YAP subcellular localization was not affected and was predominantly distributed in the nuclear of cells on all substrates (Figure 5(a)). As the substrates became stiffer, the YAP level of MC3T3-E1 increased dramatically (Figure 5(b)). These data indicated that YAP expression level is regulated by matrix stiffness, but stiffness range designed here did not change YAP localization. The mRNA level of RAP2, a mediator of the Hippo pathway responding to matrix stiffness, was also evaluated. As indicated in Figure 5(c), there were no significant differences among the mRNA levels of RAP2 in cells on the four substrates.

## 4. Discussion

Understanding the relationship between cell behaviors and the implant surface property aids in ensuring the device's effectiveness. The osteogenic differentiation of stem cells can be artificially regulated by modifying their microenvironments. Previously, a number of studies have shown that stem cells differentiate in response to varying substrate stiffness [3, 20]. Matrix stiffness has a profound effect on cell fate decision [6, 21, 22], and specific stiffness can contribute to stem cell fate [5, 23]. However, whether osteoblast differentiation is mediated by specific stiffness is unclear.

Previous studies examined stem cell behavior on a range of PDMS-based matrices with degrees of stiffness from ~1 kPa to ~100 kPa, and results showed that stiffer matrix upregulates the marker of osteogenesis compared with cells on softer matrices [6, 11]. To date, researchers agreed that stiff substrates are favorable for osteogenic differentiation of cells, whereas soft matrices promote adipogenic differentiation [11, 21], but little is known about how to precisely control the osteogenic lineage. A high level of osteogenic differentiation may be present in the cells on a stiff matrix. To explore the differentiation ability of MC3T3-E1 on matrices with tunable stiffness, we fabricated four polymer substrates with varying stiffness. Sylgard 184 has been used in cell culture studies frequently, and the common approach is to obtain substrates with different stiffness to decrease the ratio of curing agent to base resin [24]. However, the ratio of 1:10 from the manufacturer's recommendation is the optimal stoichiometry of the cross-linking reaction. Altering the amount of crosslinker may lead to the formation of noncross-linked PDMS layer on the surface [25], which may affect cell adhesion or other behavior [26], and mask the effects of surface stiffness. Here, substrates were fabricated with Sylgard 184 to Sylgard 527 ratios of 1:0, 5:1, 1:1, and 1:3 to produce a range of matrix stiffness from 2.7 MPa to 0.6 MPa. This method maintains the stoichiometry of the individual PDMS types, and substrate modulus can be controlled independent of the surface properties, including roughness, energy, and protein adsorption [12]. The data of elastic modulus tested in this study are higher than those of the previously reported results [12]. This finding does not indicate the unreliability of our results. Existing results from different work also show that the elastic moduli of the standard Sylgard 184 formulation with 1:10 curing agent to base ratio range from 1 MPa to 2.5 MPa [24, 26, 27]. The causes of the variability in different reports remain unclear; the difference in curing time and temperature may be one of the main factors [12].

The expression levels of key osteogenic markers, including OCN, Col I, OPN, and calcium nodule, were detected. OCN, Col I, and OPN serve as late dominant markers of differentiated osteoblasts. Col I is the abundantly present organic component in bone (about 90%) [28]. OCN is synthesized by mature osteoblast and primarily deposited in the ECM of bones. OCN and OPN play a role in the mineralization of bone and are involved in osteogenic differentiation. They are frequently used as markers of osteogenic differentiation [16, 29]. The MC3T3-E1 commitment to osteoblast was enhanced with decreasing ECM stiffness. This result suggested that less stiff substrates in our study are more conducive to osteoblastic differentiation than stiffer substrates. Substrates with the ratio of 1:3 resulted in the highest osteogenic phenotype levels among the matrices. The range of elastic modulus we considered was much larger than that reported in the literature. Our experiments showed that it is not simply “the stiffer, the better.” By contrast, for matrix stiffness much greater than that of in situ osteoid [11], softer matrix is more inductive for osteogenesis of cells than stiffer ones. Engler et al. [6] reported that the multipotent differentiation of stem cells is sensitive to tissue-level stiffness. Together with previous observations, we conclude that there should be an optimal substrate stiffness that can promote osteogenic differentiation distinctly, and it is not simply “downregulated in softer substrates and upregulated in stiffer substrates.”

Studies also revealed a definitive interplay between the substrate stiffness and cell adhesion or morphology [11, 30]. Alterations in substrates stiffness may cause changes in cell adhesion [30]. Cells sense the substrate stiffness through adhesion sites and respond by altering their cytoskeletal structure, which in turn affects their adhesion to the substrate. The morphology of adherent cells on matrix may influence cell survival and differentiation. Some studies have demonstrated that substrate stiffness affects cell spreading area [6, 31]. Other reports also showed that stiffness had no effect on cell morphology [32, 33]. Here, our results indicated that stiffness had no effect on cell spreading area or growth of MC3T3-E1 on our polymer networks, although we observed changes in the expression of differentiation markers. The consistency in cell shape may be due to the fact that the distribution of cell binding domain is independent of the stiffness range designed in this paper. Cells reached their maximum spreading area on all substrates and the growth of cells was not affected. Thus, a lack of correlation between cell spreading and matrix stiffness may be observed [33, 34]. Possibly, it can be understood that differentiation process does not necessarily correlate with outward changes in cell morphology. Mechanical stimuli can be transferred from surface adhesive proteins into cell body [32]. FA contributes to cell adherence [30]. Vinculin is a key FA protein and serves as an essential link between external physical stimuli and cytoskeleton. In the present study, vinculin was stiffness-dependent; that is, rigid matrices allowed strong vinculin expression in cells, which was in agreement with the results of a previous report [11]. It also suggested that the adhesion of MC3T3-E1 cells was increased with increasing the stiffness of substrates. Our results are consistent with a recent study [30]. Previous studies reported that enhanced level of vinculin upregulates cellular functions, such as cytoskeletal structure and differentiation [14–16]_._ However, our data showed that the vinculin level of MC3T3-E1 was decreased as the substrate stiffness decreased, which was in contrast to the results of osteogenic differentiation markers. In other words, increased cell adhesion may not positively promote the osteogenic ability of cells. We suppose that decreased vinculin did not influence the main process of stiffness-dependent osteogenesis in this study. Other signaling molecules, such as integrins, may have mediated the stiffness-sensing mechanisms of cells [35]. Therefore, more binding mechanisms should be addressed. The overexpression or knockdown of vinculin should be tested for its effect on MC3T3-E1 differentiation in the future.

Intracellular signals generated following stiffness stimulation have not been elaborated well. Hippo signaling has been demonstrated to be linked to the nuclear transduction of mechanical signals [36]. YAP is one of the essential transducers of the Hippo pathway. Our previous study showed that YAP may play a role in grid topology substrates-induced MC3T3-E1 differentiation and is associated with the substrate-based control of cell biological behaviors [16]. Literature suggests that YAP is a nuclear relay of mechanical signals exerted by substrate stiffness [18]. YAP is clearly nuclear on stiff substrates but becomes predominantly cytoplasmic on soft substrates [37]. YAP/TAZ activity and subcellular localization are regulated by ECM stiffness. Dupont et al. reported that YAP/TAZ were clearly nuclear on hard substrates (15-40 kPa) but became predominantly cytoplasmic on softer substrates (0.7-1 kPa) [18]. That is to say, YAP/TAZ activity and subcellular localization are regulated by ECM stiffness. Matrix with tunable stiffness here may regulate YAP expression or localization. Therefore, we got immunocytochemistry and WB data of YAP. Consistent with a previous study [18], our data demonstrated that MC3T3-E1 cells seeded on our substrates exhibited low YAP level on soft surfaces. Notably, YAP is predominantly nuclear in MC3T3-E1 on all tested substrates. The subcellular location of YAP is mostly dependent on cell morphology. Combined with the reported conclusions, we deduced that YAP is predominantly nuclear as long as the elastic modulus of the substrate is >40 kPa [18]. It has been reported that enhanced YAP level of cells remarkably promotes osteogenesis, and osteogenic differentiation induced in MSC on stiff substrate is inhibited upon YAP depletion [38, 39]. However, the data in the present study showed that YAP expression decreased with the gradual decrease in the stiffness of substrates, but the osteogenesis degree increased. A recent report identified the Ras-related GTPase RAP2 as a key intracellular signal transducer that relays matrix rigidity signals to control mechanosensitive cellular activities through YAP and TAZ [40]. They confirmed that RAP2 is activated by low ECM stiffness, and deletion of RAP2 blocks the regulation of YAP and TAZ by stiffness signals and promotes aberrant cell growth. So we further performed the expression of RAP2 of MC3T3-E1 cells on our substrates with different stiffness. Result showed that there were no significant differences among the mRNA levels of RAP2 in cells on the four substrates. Although we do not have enough data to elaborate the signaling pathway well, we hypothesized that osteogenic differentiation process may be independent of YAP signaling in the range of stiffness with elastic moduli 0.6 MPa to 2.7 MPa. The changes in stiffness that did not trigger RAP2 activity or YAP to shift to the cytoplasm may also be a reason why the reduction in YAP expression did not decrease the cell osteogenic differentiation on the substrates. This is an important point and we are going to explore further in the following work. Our study has several limitations. The relationship between YAP and cell differentiation requires further study, particularly the molecular mechanisms involved. In addition, the osteogenic differentiation of MC3T3-E1 on surfaces with a wider range of elastic modulus may be analyzed, and the results may enhance the understanding of the bone differentiation processes and determine the optimal stiffness to promote osteogenesis.

## 5. Conclusions

Substrate stiffness affects the osteogenic differentiation of MC3T3-E1 without affecting cell morphology. A soft matrix (with low stiffness, 1:3) stimulates the process of osteogenic differentiation of MC3T3-E1 cells than substrates with higher stiffness (1:1 to 1:0). For matrix stiffness much greater than that of in situ osteoid, the osteogenic differentiation of MC3T3-E1 increased with decreasing substrate stiffness. Meanwhile, YAP was downregulated. Hence, the mechanism may be independent of YAP signaling. These results provide a theoretical basis in the control of cell fate, which is an important consideration in the design of biomaterials used for tissue repair.

## Data Availability

The figure and table data used to support the findings of this study are included within the article.
